# Pulsed Electric Field Pretreatment Enhances the Enzyme Hydrolysis of Baker’s Yeast

**DOI:** 10.3390/microorganisms12122470

**Published:** 2024-12-01

**Authors:** Ralitsa Veleva, Valentina Ganeva, Miroslava Zhiponova

**Affiliations:** 1 Department of Cell Biology and Developmental Biology, Faculty of Biology, Sofia University “St. Kliment Ohridski”, 1164 Sofia, Bulgaria; ralitsa_veleva@biofac.uni-sofia.bg; 2 Department of Biophysics and Radiobiology, Faculty of Biology, Sofia University “St. Kliment Ohridski”, 1164 Sofia, Bulgaria; 3 Department of Plant Physiology, Faculty of Biology, Sofia University “St. Kliment Ohridski”, 1164 Sofia, Bulgaria; zhiponova@biofac.uni-sofia.bg

**Keywords:** pulsed electric field, irreversible electropermeabilization, Alcalase, enzyme hydrolysis yeast extract, antioxidant activity, peptides

## Abstract

Baker’s yeast is a key starting material for producing extracts with diverse compositions and applications. This study investigates the effect of pulsed electric field (PEF) pretreatment, which induces irreversible electropermeabilization, on the enzymatic hydrolysis of yeast. Cell suspensions were exposed to monopolar rectangular pulses in a continuous flow system followed by 4 h of incubation with Alcalase at concentrations of 0.2% and 0.5%. PEF pretreatment significantly improved enzymatic hydrolysis, with maximum intracellular content recovery under electrical conditions resulting in outlet temperatures of 56–58 °C. The released protein reached 163.7 ± 13 mg per gram of dry cell weight (DCW). SDS-PAGE analysis showed that the extracts predominantly contained peptides with molecular masses below 4.7 kDa. The phenolic content was comparable to that of cell lysates obtained after mechanical disruption. The free α-amino nitrogen content and total antioxidant activity reached 218.2 ± 26 mg/gDCW and 53.4 ± 4.6 mg TE/gDCW, respectively, representing 3.2-fold and 2.65-fold increases compared to cell lysates. The hydrolysates from PEF-pretreated cells demonstrated a positive effect on the proliferation of the human keratinocyte cell line HaCat. The obtained data lead to the conclusion that PEF pretreatment is a promising approach to enhance the production of yeast hydrolysates with various applications.

## 1. Introduction

Baker’s yeast (*Saccharomyces cerevisiae*) is the most widely distributed commercial yeast worldwide. Beyond its primary use in baking and fermentation processes, such as wine and beer production, *S. cerevisiae*—which holds GRAS (Generally Recognized As Safe) status—is a rich source of proteins, essential amino acids, vitamins, minerals, antioxidants, and other bioactive compounds. Yeast cell biomass can be utilized in various forms, such as whole cells after inactivation by heating, as autolysates or hydrolysates, or after extraction, fractionation, and purification of the valuable intracellular compounds which find diverse applications across agriculture, food, cosmetics, and pharmaceutical industries [1,2,3].

Baker’s yeast is a key starting material for the production of yeast extracts with various compositions and applications. The two main methods for obtaining yeast extracts are (i) autolysis, which involves the digestion of cell wall components and intracellular macromolecules by endogenous enzymes, a process that can be optimized with the addition of salts or organic solvents, and (ii) enzymatic hydrolysis using exogenous enzymes [4,5,6,7].

An important characteristic of the yeast extracts is their antioxidant activity. In addition to the cell wall polysaccharide components (β-glucan and mannan) possessing antioxidant properties, yeast contains various intracellular antioxidants, including antioxidant enzymes and a range of low-molecular-weight antioxidants like glutathione, amino acids, vitamins, and phenols [8,9,10]. In recent years, there has been growing interest in obtaining yeast extracts enriched with peptides (short chains of amino acids with usually 2–20 amino acid residues) that exhibit diverse biological activities. Depending on their size, composition, and physicochemical properties, these peptides enhance the antioxidant activity of the extracts and confer antimicrobial, antihypertensive, and immunomodulatory properties [11,12,13].

One of the most efficient methods for obtaining yeast extracts with a high content of biologically active peptides is enzymatic hydrolysis, using either a single exogenous endopeptidase or a mixture of enzymes (endo- and exopeptidases, β-glucanase) applied simultaneously or sequentially. Enzymes such as trypsin, chymotrypsin, papain, and Flavourzyme^®^ are commonly used, and in the last years, data on the production of yeast hydrolysates from brewer’s and baker’s yeast using Alcalase^®^ also appeared [5,7,11,14,15,16]. Alcalase is a serine endopeptidase with a molecular weight of 27.3 kDa and is derived from *Bacillus licheniformis*. It is commonly applied in the production of protein hydrolysates with antioxidant properties from various sources [17,18]. Due to the wide range of amino acids that the enzyme can recognize, the reaction of the protein hydrolysis catalyzed by Alcalase has a strong tendency to give a hydrolysate with many small peptides.

Enzymatic treatment is applied directly to intact fresh cells or cells subjected to different pretreatments, such as freeze drying or heat treatment. The release of significant intracellular content from commercial baker’s yeast usually requires prolonged incubation (12–48 h) with the enzymes at temperatures optimal for their activity, ranging between 50 and 60 °C [5,7,15,19]. The primary reason for this is the presence of the cell envelope, the plasma membrane and the cell wall, which acts as the main barrier hindering the extraction of intracellular components [20]. The yeast cell wall is an elastic, layered structure, 100 to 200 nm thick, that protects the cell from osmotic swelling and physical stress [21]. In stationary-phase cells, the cell wall becomes thicker and more rigid, significantly reducing its permeability to macromolecules and impeding the access of added enzymes to the cell interior. Prolonged incubation at relatively high temperatures, necessary to induce partial digestion of the cell wall and destabilization of the plasma membrane, can alter the structure of the resulting peptides and diminish their biological activity, including the antioxidant properties [12,22].

To enhance the access of exogenous enzymes to intracellular proteins and shorten the time required for efficient hydrolysis, a treatment can be applied to permeabilize the plasma membrane and increase the porosity of the cell wall. In recent years, pulsed electric field (PEF) treatment has gained popularity as a fast, non-destructive, and scalable method for the permeabilization of microorganisms and plant cells, allowing the extraction of various intracellular components [23,24]. The electrical treatment induces a change in plasma membrane integrity, known as electropermeabilization or electroporation, by generating an additional transmembrane potential [25,26]. Depending on the electrical parameters, pulsing media composition, cell concentration, and the post-pulse incubation condition, the loss of membrane barrier functions can be irreversible (irreversible electropermeabilization), resulting in a massive release of intracellular content [27,28,29,30].

A significant advantage of a moderate PEF is that it generally does not cause fragmentation of cells with cell walls. This allows for the selective extraction of intracellular components based on their size and charge, their location in different compartments, and their hydrophobicity [31,32,33,34]. A PEF applied in flow mode has been successfully used for the extraction of small water-soluble molecules, such as free amino acids, antioxidants, and nucleotides, as well as proteins both native and recombinant from different yeast species [28,29,34,35,36,37,38]. It has been shown that electrical treatment leading to an efficient protein recovery not only provoked irreversible permeabilization of the plasma membrane but also enhanced the cell wall porosity, thus enabling the release of even very large intracellular and periplasmic enzymes from yeast [39,40].

A PEF, which induces irreversible electropermeabilization, also allows the entry of various compounds, including macromolecules, into cells with cell walls when they are added to the suspensions after electrical treatment. Recently, it has been demonstrated that PEF pretreatment promotes the enzymatic hydrolysis of fresh biomass from the microalga *Scenedesmus almeriensis* by enabling the entry of the enzymes Alcalase and Flavourzyme into the cells [41].

In this study, we evaluate, for the first time, the potential of PEF pretreatment of yeast cells, followed by their incubation with an exogenous protease, as an alternative method for producing yeast hydrolysates with diverse potential applications.

## 2. Materials and Methods

### 2.1. Yeast Strain

The experiments were conducted using commercial fresh baker’s yeast (VIVO, Lesaffre Bulgaria Ltd., Sofia, Bulgaria). The cells were washed once with distilled water (2600× *g* for 10 min), resuspended in distilled water, and incubated for 1 h. Following incubation, the suspension was centrifuged, and the cells were diluted in distilled water to a final concentration of 57 g dry cell weight per liter (gDCW/L). The conductivity of the suspension was then adjusted to 200 ± 5 µS/cm using a 0.25 M sodium phosphate buffer at pH 7.

### 2.2. Pulsed Electric Field (PEF) Treatment

The electric field treatment in a continuous-flow chamber was performed with a generator of monopolar rectangular pulses (2300V-10A) and a Hydropuls mini (GBS-Elektronik, Radeberg, Germany) as already described [37]. The pulse duration and frequency were regulated by an arbitrary waveform generator RIGOL DG1012 (Suzhou, China). The chamber (0.825 mL volume) has two parallel stainless steel electrodes that are 0.4 cm apart. The PEF treatment was performed at flow rates of 35 mL/min, controlled by a peristaltic pump (Ismatec, Glattbrugg, Switzerland). During the passage through the chamber, the cells received 20 pulses with a duration of 0.5 ms (total treatment time 10 ms) and electric field strength in the range of 3–3.7 kV/cm. All pulsing parameters were monitored online with an Instek GDS 2064 oscilloscope (New Taipei City, Taiwan). The inlet temperature of the suspensions was 21–22 °C. The outlet temperature was registered by a K-type thermocouple connected to a digital thermometer, and the sensor was attached to the end of the tubing. To induce irreversible plasma membrane permeabilization in this study, cell suspensions were treated in continuous flow mode with 20 pulses of 0.5 ms duration and field strength ranging from 3 to 3.7 kV/cm. At a flow rate of 35 mL/min, the residence time of the cells inside the pulsing chamber was about 1.4 s, and the outlet temperature was registered about 4 s after that. Enzyme hydrolysis was then performed using cell suspensions treated at two different field strengths, both leading to over 90% irreversibly permeabilized cells: field strength of 3.3 kV/cm, leading to outlet temperatures of 46–48 °C, and field strength of 3.65 kV/cm, leading to outlet temperatures of 56–58 °C.

### 2.3. Determination of Irreversible Electropermeabilization

Membrane permeabilization was assessed by staining the cells with propidium iodide (PI) [39]. To determine the fraction of cells with irreversibly permeabilized membranes, 5 µL of a 0.5 mM PI solution in distilled water was added to 50 µL of cell suspension 1 h after pulsing. The number of fluorescent cells was then counted using an epifluorescent microscope (L3201 LED, Microscopesmall, Shenzhen, China). Permeabilization was expressed as the percentage of fluorescent cells relative to the total cell count.

### 2.4. Treatment with Alcalase

Suspensions of control and electrically treated cells were adjusted to an initial pH of 8.5 by adding 2N NaOH. Then, 10 mL of each cell suspension was transferred into 50 mL Falcon tubes and incubated for 15 min at 48 °C. Alcalase^®^ 2.4 L (Novozymes, Bagsværd, Denmark) was added to achieve final concentrations of 0.2% (*v*/*v*) and 0.5% (*v*/*v*). The cell suspensions were then incubated for 4 h at 48 °C with shaking at 100 rpm in a Biosan ES-20/40 orbital shaker. By the end of the incubation, the pH of the electrically treated cell suspensions was 7.5. The pH of the control suspensions was maintained at the same level during incubation by adding NaOH. After 4 h of incubation with Alcalase, the suspensions were centrifuged for 10 min at 2600× *g*, and the extracts were transferred to clean tubes. The cells were washed once with distilled water, and the resulting supernatants were adjusted to a final volume of 25 mL. A portion of the supernatants was then heated for 15 min at 90 °C and centrifuged at 13,400 rpm for 5 min, and the supernatants were transferred to clean tubes. All supernatants were stored at −20 °C for later use.

### 2.5. Preparation of the Cell Lysate

A total of 1 mL of a water suspension of control and untreated cells was mixed with an equal volume of glass beads (diameter 0.42–0.6 mm, Sigma-Aldrich Chemie GmbH, Schnelldorf, Germany) and vortexed 9 times for 1 min each, with a 15 s pause on ice between intervals [37]. The resulting lysate was centrifuged at 10,750× *g* for 5 min. This procedure leads to about 99% cell lysis, as determined by counting the cells in a Thoma chamber. The supernatant was transferred to a clean tube, and the pellet was washed once with distilled water. The extracts were adjusted to a final volume of 2.5 mL and stored at 4 °C until the evaluation of the total soluble protein, free α-amino nitrogen, total antioxidant activity, and phenolic content.

### 2.6. Analytical Methods

Total protein was determined according to Lowry et al. [42] with bovine serum albumin as a standard. Free α-amino nitrogen was determined with the Ninhydrin method according to Lamoolphak et al. [43] with leucine as a standard.

Total antioxidant activity was determined by TEAC (Trolox equivalent antioxidant capacity) assay using 2,2′-Azino-bis (3-ethylbenzothiazoline-6-sulfonic acid) diammonium salt (ABTS) as described by Pellegrini et al. [44] with slight modifications. The ABTS•+ stock solution (7 mM aqueous solution of ABTS with 2.45 mM potassium persulfate) was diluted with 50 mM potassium phosphate buffer pH 7 to the absorbance of 0.70 ± 0.02 at 734 nm. The samples (5 µL) of two-fold diluted water extracts were mixed with 995 µL of working solution. The decrease in the absorbance at 734 nm was measured after 15 min. The ABTS•+ scavenging activity was calculated as follows: scavenging effect (%) = (1 − A1/A0) × 100%, where A1 was the absorbance of the sample and A0 was the absorbance of the blank control. The Trolox standard curve was determined in the range of 0.1 to 15 µM. The data are expressed as mg Trolox equivalent per gram of cell dry weight—mgTE/gDCW.

The total phenolic content was determined according to Singleton et al. [45] with modifications. Test samples contained 0.1 mL supernatant of electrically treated and control cell suspensions or cell lysate, 1.5 mL Folin–Ciocalteau reagent (previously dissolved in distilled water 1:10), and 1.4 mL 7.5% sodium carbonate. The samples were incubated in the dark at room temperature for 30 min. The absorbance was measured at λ = 765 nm by a Shimadzu UV 1800 spectrophotometer. A standard curve, made with known concentrations of gallic acid (GA), was used to calculate the content of phenolic compounds per g DCW.

The Tricine-Sodium dodecyl sulfate polyacrylamide gel electrophoresis (Tricine-SDS-PAGE) of the protein samples was performed on 14.7% polyacrylamide slab gels, as described by Schägger [46]. Proteins were detected by silver staining, following the method of Nesterenko et al. [47], and the protein molecular weight markers used were PageRuler™ Prestained Protein Ladder (1–40 kDa).

The yeast biomass was measured as dry cell weight (DCW) using an infrared moisture analyzer method according to Li and Mira de Orduña [48].

### 2.7. Determination of Cell Viability and Cell Cycle of Human Keratinocyte Cell Line HaCat

Cells were routinely cultivated in 90 mm Petri dishes before experiments and incubated under standard conditions: 5% CO_2_ and 37 °C in DMEM supplemented with 10% FBS and 1% (*v*/*v*) antibiotic–antimycotic solution (penicillin 100 U/mL, streptomycin 100 μg/mL and amphotericin B 0.25 μg/mL).

For this study, the extract was obtained by incubating PEF-treated yeast cells (3.65 kV/cm) with 0.5% Alcalase. To inactivate the enzyme, the extract was heated to 90 °C, as explained above. Then, the extract was freeze dried for 24 h, diluted in DMEM to a final protein concentration of 2.23 mg/mL, and filtered through a 0.2 µm Durapore filter (Millipore Co., Bedford, MA, USA).

Cell viability was estimated through crystal violet assay [49]. Cells were seeded in 96-well plates with a concentration of 20 × 10^4^ cells per mL for these experiments. They were incubated for 24 h to recover their morphology, cell contacts, and cell cycle after trypsinization. After 24 h, the media were carefully removed and replaced with culture media supplemented with extract to a final concentration of protein between 0.139 and 2.23 mg/mL. Twenty-four hours after treatment, the samples were washed with PBS and fixed with 4% buffered formaldehyde solution for 20 min. Cells were washed with distilled water and stained with 1% crystal violet solution for 20 min. The samples were washed and air dried. We used 10% acetic acid to dissolve the dye. The optical density of the samples was measured at 570 nm by Epoch Microplate Spectrophotometer (BioTek, Winooski, VT, USA) with Gen5™ Data Analysis software, version 1.11.5. The results are presented as a percentage of cell viability compared to the control untreated cells.

To study the effect of the extract on the cell cycle, the cells were seeded in 6-well plates with concentrations of 10 × 10^4^ cells per mL for these experiments. Samples were incubated with DMEM supplemented with extract to final concentrations of protein of 0.573 mg/mL and 1.147 mg/mL. At 24 and 48 h after treatment, the cells were washed with PBS, trypsinized, and centrifuged, the supernatant was discarded, and the cells were resuspended in PBS and centrifuged again. The cells were fixed in 70% ice-cold ethanol overnight and stained with Guava Cell cycle reagent [50]. The data for the cell cycle were obtained on the Guava easy Cyte instrument. Each cell is distributed in one of the cell cycle phases according to its DNA content.

### 2.8. Statistics

The results from three to four independent experiments are shown as a mean value ± SD. Student’s *t*-test was applied for statistical evaluation. Differences were considered significant if *p* < 0.05. Part of the data was analyzed with OriginPro 9.0 and presented as a mean value ± SD. Statistical significance is according to one-way ANOVA at the 0.05 level.

## 3. Results and Discussion

Alcalase is one of the most effective commercial enzymes used for the hydrolysis of proteins from various sources. Due to its relatively low molecular weight, we hypothesized that this enzyme could more easily pass through the cell wall and reach the intracellular space. To facilitate enzyme entry, the applied electrical treatment must induce irreversible permeabilization of the plasma membrane in a significant percentage of the cells. Under such conditions, the electric field increases also cell wall permeability [37,40].

Although PEF treatment is considered a non-thermal method, under certain conditions, it can provoke a significant temperature rise due to Joule heating. This phenomenon results from a strong increase in conductivity during pulse application, which is caused by the rapid release of intracellular ions. In such cases, when the PEF is applied in continuous flow mode, the cells experience a brief thermal shock as they pass through the pulsing chamber. Treating cell suspensions of baker’s yeast under electrical conditions where outlet temperatures range between 46 and 49 °C does not lead to protein denaturation. On the other hand, when the outlet temperature exceeds 51 °C, changes in the conformation of soluble intracellular proteins and mannoproteins forming the outer cell wall layer are induced, resulting in partial enzyme inactivation, lower sensitivity of the cells to lytic enzyme treatment, and reduced efficiency of protein extraction [37].

The aim of our study was to investigate two potential effects of the electric field on the efficiency of enzymatic hydrolysis: (i) the loss of membrane barrier function (electropermeabilization) and the increased permeability of the cell wall to macromolecules and (ii) structural changes in proteins (both soluble and those associated with the cell wall) induced by a brief thermal shock during the passage of cells through the pulsing chamber. That is why enzyme hydrolysis was performed using cell suspensions treated at a field strength of 3.3 kV/cm, leading to outlet temperatures of 46–48 °C, and a field strength of 3.65 kV/cm, leading to outlet temperatures of 56–58 °C. At both electrical conditions, the percentage of irreversibly permeabilized cells was over 90%.

### 3.1. Protein Release

Typically, the analysis of released protein after enzymatic hydrolysis is performed after heating the samples to inactivate the hydrolytic enzymes used. Unlike peptides (molecular mass < 5 kDa), proteins with a molecular mass greater than 10–15 kDa tend to unfold and precipitate during heat treatment. Therefore, in this study, total protein content determination and Tricine-SDS-PAGE analysis of the extracts were performed using non-tempered samples, as free Alcalase is practically inactive at room temperature [51]. Measurements were taken immediately after thawing the samples, and the results were adjusted against controls containing the same concentration of Alcalase.

**Figure 1:**
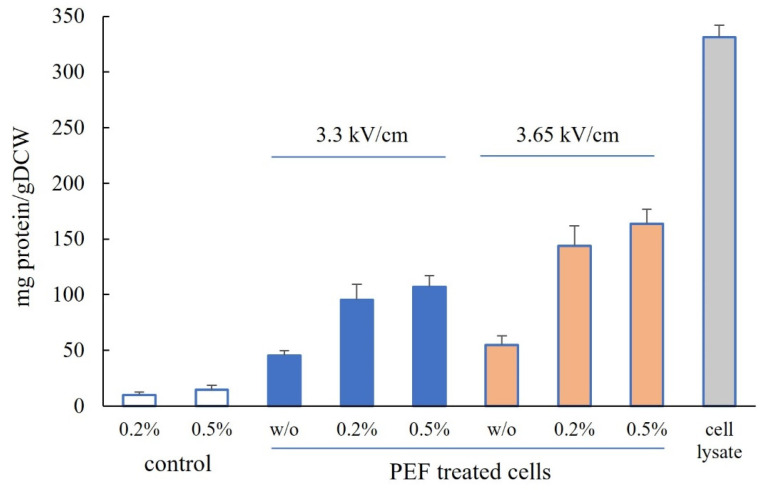
Protein content of cell lysates obtained after mechanical disruption and extracts obtained from control cells and cells subjected to PEF treatment at 3.3 kV/cm and 3.65 kV/cm after incubation for 4 h at 48 °C with or without (*w*/*o*) Alcalase. The values represent the mean ± SD derived from three independent experiments.

As shown in Figure 1, incubation of the control (PEF untreated) cells with Alcalase resulted in the release of only 9.8 to 14.4 mg of protein per gram of DCW.

The protein content of extracts from PEF-treated cells incubated without Alcalase ranged from 45 to 50 mg protein/gDCW. There is evidence that PEF treatment can indirectly accelerate autolysis due to osmotic imbalance, vacuolar lysis, and the liberation of endogenous hydrolytic enzymes; however, this is a more prolonged process [38,52]. In our protocol, the incubation of the cells at 48 °C was limited to only 4 h, which is too brief for significant activation of endogenous proteases. Therefore, these are most likely intracellular peptides released due to the irreversible permeabilization of the plasma membrane.

On the other hand, PEF pretreatment significantly enhanced the efficiency of the enzyme hydrolysis. The protein content of the extracts reached values 2.5–3 times higher than that obtained from pulsed cells incubated without enzyme. A higher protein yield was obtained from cells treated at 3.65 kV/cm, where outlet temperatures reached 56–58 °C, suggesting that partial protein denaturation may influence hydrolysis efficiency. Depending on the Alcalase concentration, the protein content reached 143.7 ± 18 and 163.7 ± 13 mg protein/gDCW, which corresponds to 43% and 49% of the total soluble protein released after mechanical disruption, respectively. Incubation with Alcalase leads to the release of a significant amount of protein from electrically treated cells in a relatively short time, suggesting that the enzyme can readily cross the cell wall and reach the cell interior without requiring additional treatments, such as incubation with a reducing agent or lytic enzymes, to increase cell wall porosity.

This effect can be partly attributed to the enzyme’s relatively low molecular weight, which likely facilitates its passage through the cell wall and is further aided by an electroinduced increase in cell wall porosity. Additionally, the brief thermal shock experienced by the cells as they pass through the pulsing chamber may induce partial denaturation of cell wall mannoproteins, thus making them more susceptible to enzymatic hydrolysis, which could further enhance the permeability of the wall.

**Figure 2:**
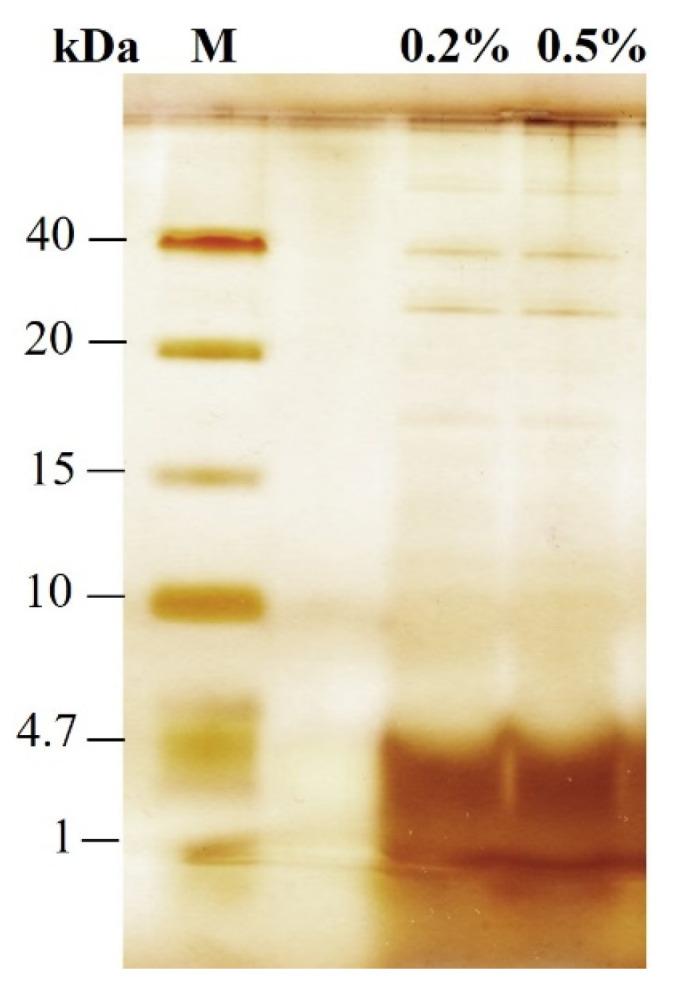
Tricine-SDS-PAGE analysis of the extract obtained from PEF-treated cells (3.65 kV/cm) after 4 h of incubation with 0.2% and 0.5% Alcalase.

Analysis of the supernatants using Tricine-SDS-PAGE shows that the extracts obtained from PEF-treated cells incubated with Alcalase contain primarily peptides with a molecular mass below 4.7 kDa (Figure 2). There are only traces of proteins with molecular masses in the range of 20–40 kDa and possibly higher molecular weight proteins that remain at the start of the running gel. The presence of almost exclusively small peptides in the supernatant of electrically treated cells can be explained by the fact that unhydrolyzed intracellular proteins, as well as peptides with a larger molecular mass, are retained inside the cells by the cell wall probably because of their aggregation. This indicates that the cell wall of electroporated cells can act as a natural filter, enabling the fractionation and partial purification of peptides during the hydrolysis process.

### 3.2. Release of Free α-Amino Nitrogen

**Figure 3:**
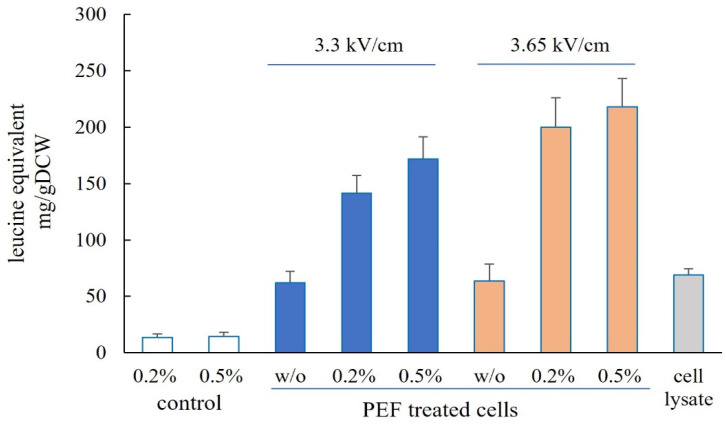
Free α-amino nitrogen (FAN) content of cell lysate and extracts obtained from control and PEF-treated cells incubated for 4 h at 48 °C with or without (*w*/*o*) Alcalase. The values represent the mean ± SD of three different experiments.

The free α-amino nitrogen (FAN) content, a parameter reflecting free amino acids and small peptides, was determined in the extracts obtained through various treatments (Figure 3). The FAN content of the cell lysate obtained after mechanical disruption was 69 ± 5.3 mg/gDCW. After incubation with 0.2% and 0.5% Alcalase, the control (PEF untreated) cells released only 13.6 and 14.4 mg/gDCW, respectively. Incubation of electropermeabilized cells with Alcalase led to a 3.16-fold increase in FAN content compared to the cell lysate and cells incubated without Alcalase. A maximum value of 218.2 ± 25 mg/gDCW was measured in extracts obtained from cells treated at 3.65 kV/cm and incubated with 0.5% Alcalase.

### 3.3. Antioxidant Activity and Phenolic Content

**Figure 4:**
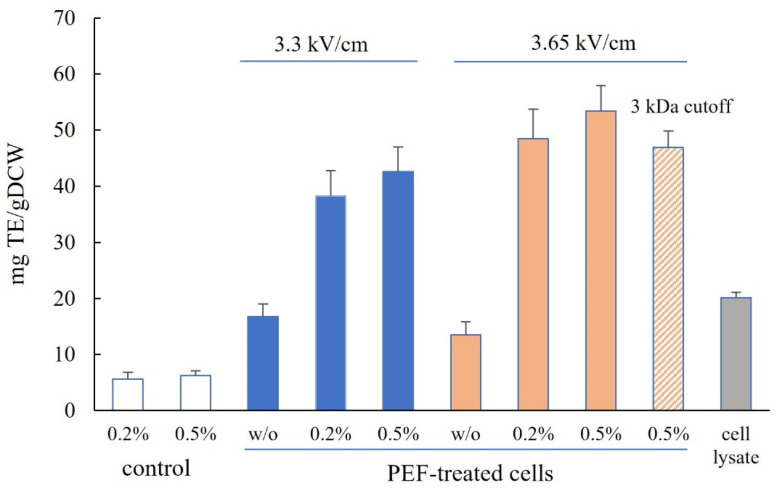
Antioxidant activity of cell lysates and extracts obtained from control cells and PEF-treated cells incubated with or without (*w*/*o*) Alcalase. The values represent the mean ± SD derived from three independent experiments.

The antioxidant activity in the cell lysate determined by the ABTS assay (Figure 4) was 20.13 ± 1 mg TE/gDCW, which is close to the value reported by Jacobs et al. [22]. The antioxidant activity of the extracts obtained from PEF-treated cells incubated without Alcalase was 16.8 ± 2.26 and 13.5 ± 2.35 mg TE/gDCW, respectively, depending on the field strength.

The incubation of PEF-treated cells with Alcalase resulted in a significant increase in the antioxidant activity of the extracts, reaching values between 1.9 and 2.65 times higher than those measured in cell lysates. The maximum antioxidant activity, 53.4 ± 4.6 mg TE/gDCW, was detected in extracts obtained from cells treated at 3.65 kV/cm and incubated with 0.5% Alcalase.

The antioxidant activity of the extracts was not affected by heating for 15 min at 90 °C or 5 min at 95 °C. Ultrafiltration with filters of varying molecular weight cutoffs (1–10 kDa) is one of the initial steps in the purification and fractionation of peptides obtained through enzymatic hydrolysis. We performed ultrafiltration on extracts from cells treated under optimal conditions (3.65 kV/cm field strength, 0.5% Alcalase incubation) using 3 kDa cutoff filters (Vivaspin 20, Sartorius, Göttingen, Germany). The antioxidant activity in the permeate showed an approximately 12% decrease, consistent with the literature indicating that the primary antioxidant activity in enzymatic yeast hydrolysates mainly originates from compounds with a molecular weight below 3 kDa [14].

**Figure 5:**
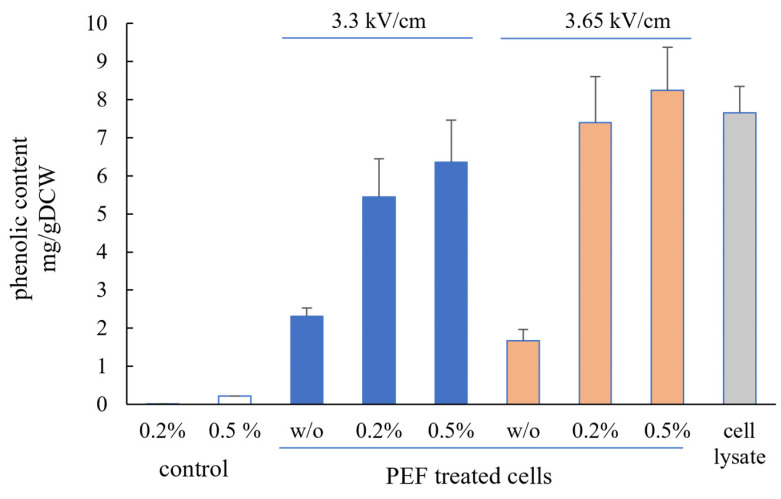
The phenolic content of cell lysate and extracts obtained from control and PEF-treated cells after 4 h incubation at 48 °C with and without Alcalase. The values represent the mean ± SD derived from three independent experiments.

Incubation of PEF-treated cells with Alcalase also significantly enhanced the phenolic content of the extracts (Figure 5).

The values obtained were 3.5 to 5 times higher than the phenolic content of extracts from PEF-treated cells incubated without Alcalase. As with other analyzed compounds, the maximum release of phenolic compounds was observed in extracts from cells treated at 3.65 kV/cm. Phenolic compounds are among the cellular components responsible for the antioxidant properties of yeast extracts. Given that the total antioxidant activity released from electroporated cells incubated with Alcalase is 1.9 to 2.65 times higher than that in cell lysates, it can be concluded that while the released phenolic compounds contribute to the antioxidant activity, they are not the primary component responsible for it. The incubation of PEF-treated cells with Alcalase similarly affects both the FAN content and the antioxidant activity of the extracts. These results, combined with the finding that the antioxidant activity is primarily attributed to compounds with a molecular weight below 3 kDa, led to the conclusion that the main contributors to the antioxidant activity in the extracts are peptides and possibly amino acids and vitamins.

### 3.4. Effect of the Extracts on Cell Viability and Cell Cycle of the Human Keratinocytes

**Figure 6:**
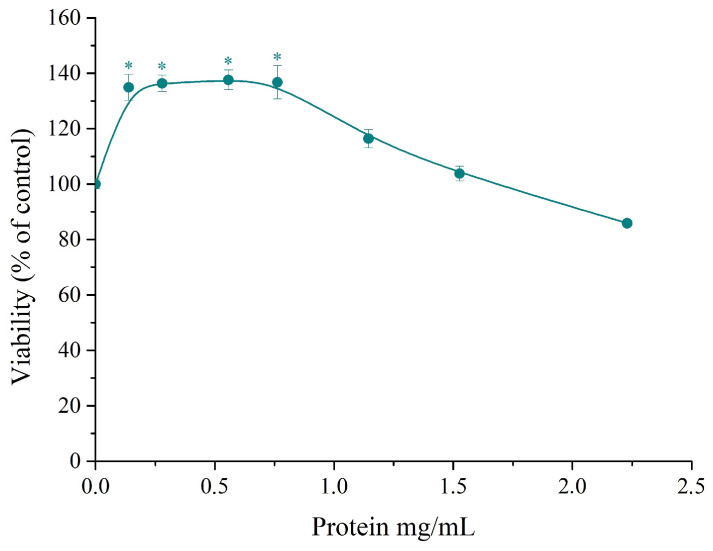
Effect of yeast extract on cell viability of HaCat cells treated for 24 h. The data are presented as a mean value ± SE derived from three independent experiments, * *p* < 0.05.

In recent years, there has been a growing interest in the use of yeast extracts in various cosmetic formulations due to their high antioxidant activity and ability to support wound healing and cell renewal [3,11,13]. In this study, we investigated the effect of the extracts on cell viability and cell cycle of the human keratinocyte cell line HaCat. The culture medium was supplemented with yeast extract to final protein concentrations ranging from 0.139 to 2.23 mg/mL. Cells were cultivated for 24 h. No cytotoxic effects were observed in media containing up to 1.53 mg/mL protein (Figure 6). Moreover, at protein concentrations between 0.139 and 0.743 mg/mL, the yeast extract increased cell viability, with the maximum effect resulting in approximately a 40% rise.

**Figure 7:**
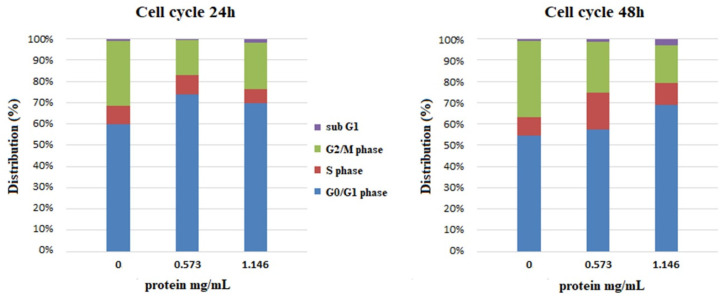
Effect of yeast extract on the cell cycle of HaCat cells treated for 24 and 48 h. The data are presented as a redistribution of cells into the different phases of the cell cycle in percentages.

To elucidate the changes in the distribution of cells in the phases of the cell cycle, samples were incubated in a medium supplemented with yeast extract to final protein concentrations of 0.573 and 1.146 mg/mL. The distribution of the cells in the phases of the cell cycle was compared with that of the negative, untreated control (Figure 7). After treatment for 24 h, there was an increase in the cell subpopulation in the G1 phase at both protein concentrations, and at a protein concentration of 0.573 mg/mL, there was a slight increase in the percentage of cells in the S phase. Prolonged treatment for 48 h led to a greater increase in the percentage of cells in the S phase at both concentrations, accompanied by a more modest increase in the G1 phase. In all samples, the cell populations with DNA content below 1n, suspectable for apoptosis, did not exceed 2.5%. The observed increase in the populations of cells in the G1 and S phases of the cell cycle along with the increase in cell viability demonstrate the potential of the obtained extracts to stimulate the proliferation of human keratinocytes.

The data obtained in this study showed that a significant increase in the concentration of all tested components at both Alcalase concentrations was achieved after treatment at a field strength of 3.65 kV/cm, where outlet temperatures reached 56–58 °C. This suggests that, in addition to irreversible plasma membrane permeabilization, the brief thermal shock experienced by the yeast cells during pulsation also enhanced the enzymatic hydrolysis.

There is substantial evidence that pretreatment causing partial protein denaturation facilitates enzymatic hydrolysis by increasing enzyme access to peptide bonds. This is particularly important for enzymes like Alcalase, which preferentially cleaves peptide bonds adjacent to hydrophobic amino acids typically located in the interior of proteins in their native conformation [53].

In addition to traditional thermal processing [54,55], various mechanical and physical pretreatments, including the PEF, have been used in recent years to enhance enzymatic hydrolysis [56]. A range of data show that PEF treatment can directly cause structural modifications of proteins, primarily affecting their secondary and tertiary structures [57,58]. This is mainly associated with the disruption of weak non-covalent bonds, such as hydrogen bonds, electrostatic, and hydrophobic interactions. Such changes are typically observed when applying very high field strength or long treatment time. These are electrical conditions, which significantly differ from those used for membrane permeabilization. Therefore, we suggest that the higher yield of various intracellular components obtained by treating cell suspensions at a field strength of 3.65 kV/cm is most likely a result of partial denaturation of cell proteins due to the thermal effect of the electric field, which facilitates enzymatic hydrolysis.

Pretreatments, such as high-pressure homogenization, bead milling, and ultrasonication, which disrupt the cell envelope (comprising the cell membrane and cell wall), are widely used for improving the access of exogenous enzymes to intracellular proteins in microorganisms [12,14,59,60]. Recently, PEF pretreatment applied in a flow mode, inducing a high degree of plasma membrane permeabilization, was shown to significantly enhance the enzymatic hydrolysis of microalgae [41]. The authors suggested that membrane permeabilization enables enzyme entry into the cells, resulting in protein hydrolysis as efficiently as in the case of freely accessible proteins after high-pressure homogenization.

Here, we provide the first data demonstrating the effect of PEF pretreatment on enzymatic hydrolysis efficiency in yeast using a commercial enzyme, specifically targeting pressed baker’s yeast. PEF treatment offers unique advantages due to its scalability and short processing time. Irreversible plasma membrane permeabilization and enhanced cell wall porosity in combination with brief heat shocks induced during continuous flow PEF treatment facilitate Alcalase access to the cell interior and increase hydrolysis efficiency. This alternative approach could enable the production of yeast hydrolysates without prolonged incubation at relatively high temperatures, which might otherwise lead to the degradation of valuable compounds. The growing interest in producing yeast extracts with potential applications in cosmetics, food, and pharmaceuticals highlights the importance and necessity of research in this area.

## 4. Conclusions

This study demonstrates the potential of PEF pretreatment to facilitate the enzymatic hydrolysis of fresh baker’s yeast, resulting in the production of extracts with high antioxidant activity after a relatively short enzyme incubation. The combination of irreversible electropermeabilization and a brief heat shock under specific electrical conditions significantly enhances enzymatic hydrolysis and improves the recovery of intracellular content. During hydrolysis, the cell wall acts as a selective filter, enabling the fractionation of peptides. The resulting hydrolysates stimulate the proliferation of human keratinocytes, even without additional purification. Further studies could optimize the individual steps of the procedure described here and confirm its scalability for the production of enzyme hydrolysates from baker’s yeast and other yeast species.

## Data Availability

The data presented in this study are available in this article.
